# Genomic characterization of *Listeria monocytogenes* and *Listeria innocua* isolated from milk and dairy samples in Ethiopia

**DOI:** 10.1186/s12863-024-01195-0

**Published:** 2024-01-31

**Authors:** Xiaoyuan Wei, Anwar Hassen, Karen McWilliams, Karen Pietrzen, Taejung Chung, Marysabel Méndez Acevedo, Tyler Chandross-Cohen, Edward G. Dudley, Jessie Vipham, Hassen Mamo, Tesfaye Sisay Tessema, Ashagrie Zewdu, Jasna Kovac

**Affiliations:** 1 Department of Food Science, The Pennsylvania State University, University Park, PA 16802 USA; 2 Department of Microbial, Cellular and Molecular Biology, College of Natural and Computational Sciences New Graduate Building, Addis Ababa University, P.O. Box 1176, Addis Ababa, Ethiopia; 3 College of Veterinary Medicine, Haramaya University, P.O. Box 138, Dire Dawa, Ethiopia; 4 Michigan Department of Agriculture and Rural Development Laboratory, East Lansing, MI 48823 USA; 5 Department of Animal Science and Industry, Kansas State University, Manhattan, KS 66506 USA; 6 Institute of Biotechnology, Addis Ababa University, P.O. Box 1176, New Graduate Building, Addis Ababa, Ethiopia; 7 Center for Food Science and Nutrition, College of Natural Sciences, Addis Ababa University, P.O. Box 1176, New Graduate Building, Addis Ababa, Ethiopia

**Keywords:** *L. monocytogenes*, *L. Innocua*, Dairy supply chain, Whole genome sequencing, MLST sequence types

## Abstract

Listeriosis caused by *Listeria monocytogenes* often poses a significant threat to vulnerable populations. Dairy products have been implicated in outbreaks of listeriosis worldwide. In Ethiopia, studies have identified *Listeria* spp. and *L. monocytogenes* in various dairy products, but the genetic diversity and phylogenetic relationships of these bacteria remain largely unknown in the low- and middle-income countries. Therefore, we conducted whole-genome sequencing on 15 *L. monocytogenes* and 55 *L. innocua* isolates obtained from different levels of the dairy supply chains across three regions in Ethiopia. Genomes were assembled and used for MLST genotyping and single nucleotide polymorphism (SNP) analysis to infer phylogenetic relationships. We identified a total of 3 *L. monocytogenes* (i.e., 2, 145, and 18) and 12 *L. innocua* (i.e., 1489, 1619, 603, 537, 1010, 3186, 492, 3007, 1087, 474, 1008, and 637) MLST sequence types among the studied isolates. Some of these sequence types showed region-specific occurrence, while others were broadly distributed across regions. Through high-quality SNP analysis, we found that among 13 *L. monocytogenes* identified as ST 2, 11 of them were highly similar with low genetic variation, differing by only 1 to 10 SNPs, suggesting potential selection in the dairy food supply chain. The *L. innocua* isolates also exhibited low intra-ST genetic variation with only 0–10 SNP differences, except for the ST 1619, which displayed a greater diversity.

**Supplementary Information:**

The online version contains supplementary material available at 10.1186/s12863-024-01195-0.

## Introduction

Listeriosis is a severe disease caused by *Listeria monocytogenes*. It primarily affects vulnerable populations, including the elderly, pregnant women, neonates, and people with compromised immune systems. Outbreaks of listeriosis in several countries have been associated with the consumption of dairy foods. For example, in Germany between 2006 and 2007, a major outbreak of listeriosis was traced back to the consumption of cheese made from pasteurized milk [1]. Another notable incident occurred in Quebec, Canada, in 2008, where a widespread outbreak of listeriosis was linked to pasteurized cheese, which affected multiple cheese retailers [2]. According to the CDC Multistate Foodborne Outbreak Notices [3], listeriosis outbreaks in the United States have been linked to various dairy products over the years. Most recently, in 2022, three listeriosis outbreaks occurred and were associated with deli meat and cheese (resulting in 16 illnesses, 13 hospitalizations, and 1 death across 6 states), Brie and Camembert cheeses (resulting in 6 illnesses and 5 hospitalizations across 6 states), and ice cream (causing 28 illnesses, 27 hospitalizations, and 1 death across 11 states). While no dairy-associated outbreaks of listeriosis in Africa have been reported, the world’s largest listeriosis outbreak (associated with ready-to-eat processed meat) was documented in South Africa [4]. Outbreaks of listeriosis demonstrate the importance of investigating the potential sources of *L. monocytogenes* within the food supply chains to mitigate contamination events. Furthermore, studies designed to understand the prevalence, distribution, and genomic diversity of non-pathogenic *Listeria* spp., such as *Listeria innocua*, can provide valuable insights into the ecology and potential sources of contamination associated with the pathogenic *L. monocytogenes*. This is particularly important in low- and middle-income countries that lack effective *L. monocytogenes* surveillance systems and therefore rely on research studies to estimate the prevalence of *Listeria* spp. and *L. monocytogenes* in the dairy supply chains. Among low- and middle-income countries in Africa, Ethiopia was estimated to have the largest number of cattle and a milk production sector with a substantial capacity to grow [5–7]. One of the factors that can contribute to the safe production of milk in Ethiopia is the effective control of dairy-associated foodborne pathogens, such as *L. monocytogenes.*

Several studies conducted in Ethiopia have identified the presence of *Listeria* spp. and *L. monocytogenes* in various milk and dairy products, including pasteurized milk, ice cream, cream cakes, cottage cheese, and yogurt [5]. The prevalence of *Listeria* spp. and *L. monocytogenes* varied among different product categories in Ethiopia. For example, in four studies involving pasteurized milk samples (*n* = 190), the prevalence of *Listeria* species ranged from 0 to 60%, while the prevalence of *L. monocytogenes* ranged from 0 to 20%. Moreover, seven studies investigating cottage cheese samples (*n* = 431) reported a *Listeria* species prevalence ranging from 0 to 53%, and a *L. monocytogenes* prevalence ranging from 0 to 5% [5].

While the prevalence of *Listeria* spp. in the Ethiopian dairy supply chain is relatively well understood, there is a gap in understanding their genomic diversity and phylogenetic relationships. Whole-genome sequencing (WGS) is one of the most reliable approaches for genetic characterization of microorganisms, including *Listeria* spp. It allows for a comprehensive analysis of nearly entire genomes, providing detailed insights into the genetic characteristics of individual isolates. Therefore, we applied WGS to investigate genetic diversity, phylogenetic relationships, and geographic distribution of *L. monocytogenes* and other *Listeria* spp. isolates collected at different levels of dairy supply chains in three regions of Ethiopia.

## Materials and methods

### Isolates and whole genome sequencing

**Fig. 1:**
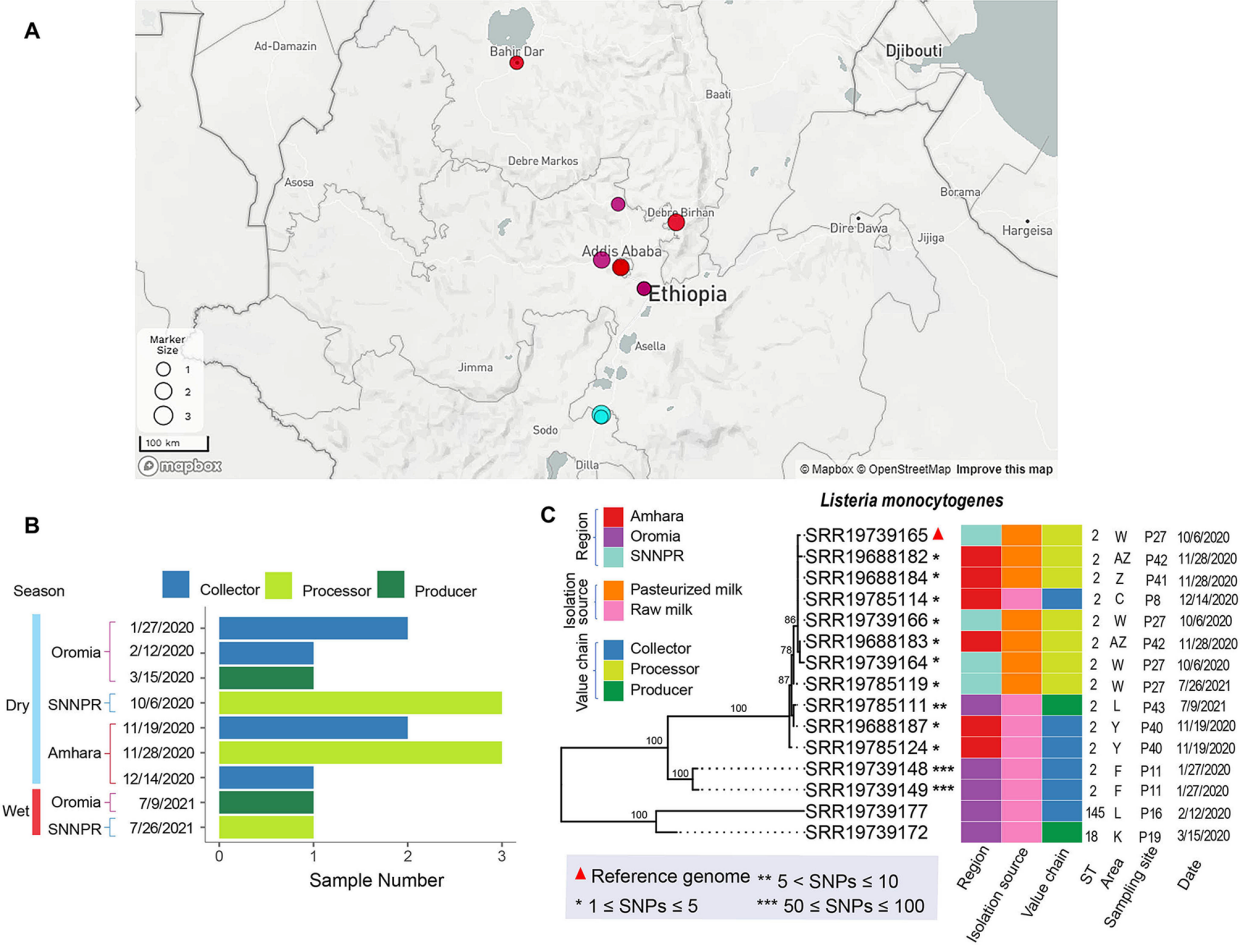
The distribution of sampling sites across three regions in Ethiopia. Each region is represented by a different color and the size of the points corresponds to the number of isolates collected at each location. Larger points indicate a higher number of isolates collected (**A**). Number of isolates (denoted as Sample Number) collected at each time point in three levels of the dairy supply chain. Different colors represent different levels within the supply chain (**B**). Maximum-likelihood phylogenetic tree of *L. monocytogenes* isolates. Each triangle represents a reference genome for a given clade, which was used to identify SNP differences among isolates. The varying numbers of asterisks (*) denote the SNP differences between each isolate and the reference genome or its respective clade. The heatmap provides additional metadata for each isolate (**C**)

**Fig. 2:**
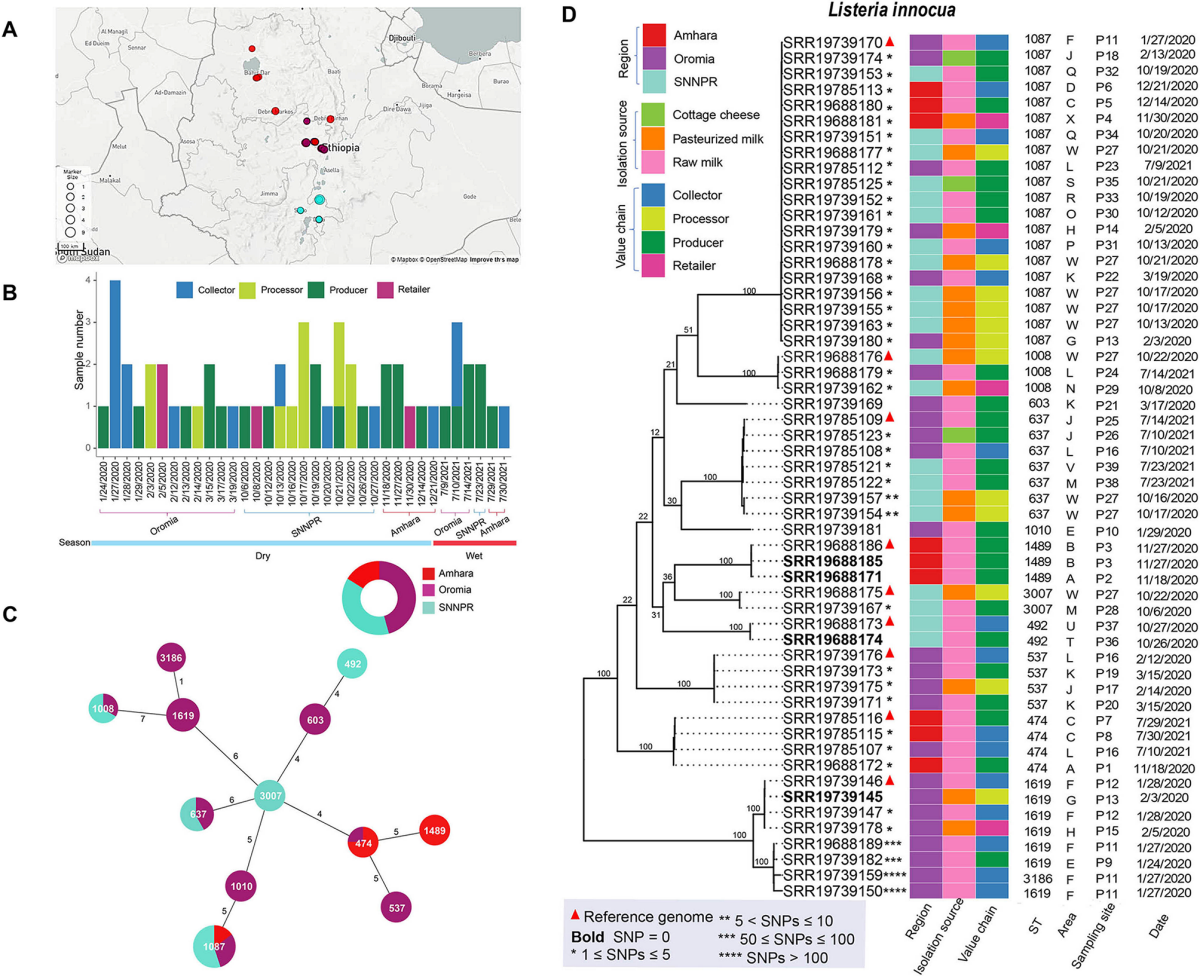
The distribution of sampling sites across three regions in Ethiopia. Each region is represented by a different color and the size of the points corresponds to the number of samples collected at each location. Larger points indicate a higher number of samples collected (**A**). Number of isolates (denoted as Sample Number) collected at each time point in four levels of the dairy supply chain. Different colors represent different levels within the supply chain (**B**). MLST-based minimum spanning tree. Node colors refer to a region of isolates’ origin. Numbers in the nodes indicate the MLST sequence types. Node sizes reflect the number of isolates with a specific sequence type. The numbers on the connecting lines illustrate the number of allelic differences (**C**). (**D**) Maximum-likelihood phylogenetic tree of *L. innocua* isolates. Each triangle represents the reference genome for a specific clade, which was used to identify SNP differences among isolates. The varying numbers of asterisks (*) denote the SNP differences between each isolate and the reference genome or its respective clade. The heatmap provides additional metadata for each isolate

Isolates characterized in this study were obtained in a previous (unpublished) study, from raw milk, pasteurized milk, and cottage cheese samples collected in three Ethiopian regions (i.e., Amhara, Oromia, and the Southern Nations, Nationalities, and Peoples’ [SNNPR]) between 2020 and 2021 (Figs. 1A and 2A). Samples were collected from 43 sites situated in 26 geographic areas, which covered four stages of a dairy supply chain, including collectors, processors, producers, and retailers (Figs. 1B and 2B). Samples were tested for *Listeria* spp., including *L. monocytogenes* by following the ISO 11290-1:2018 protocol and isolates were confirmed using a multiplex PCR [8]. Collected isolates were cryopreserved and a total of 55 *L. innocua* and 15 *L. monocytogenes* were successfully recovered for whole genome sequencing (WGS). The isolates were shipped to The Pennsylvania State University for re-confirmation and subsequently shipped to the Michigan Department of Agriculture and Rural Development Laboratory for WGS. Genomic DNA was extracted using DNeasy Blood & Tissue kit (Qiagen, Germantown, MD, USA) and the library preparation was carried out using the DNA Prep kit (Illumina, San Diego, US). The pooled libraries were whole genome sequenced using an Illumina MiSeq instrument with v2 500 cycles kit (Illumina, San Diego, US).

### Genome assembly, quality control, and taxonomic identification of isolates

FastQC (V0.11.9) [9] and Trimmomatic (V0.39) [10] with default settings were utilized to assess read quality and trim low-quality bases and adapters, respectively. Genomes were assembled using SPAdes (V3.15.4) [11] with k-mer lengths of 99 and 127, and the assembled genome quality was evaluated using Quast (V5.0.2) [12]. Contigs longer than 1000 base pairs were included in the assembly quality assessment. GTDB-Tk (V2.1.0) with the reference database version R207_v2 was used for genome-based taxonomic identification of isolates [13]. All above mentioned analyses were conducted at The Pennsylvania State University, using the supercomputer Roar.

### Multilocus sequence typing, pangenome characterization, and phylogenetic analysis

Assembled genomes were analyzed using the MLST (V2.23.0) tool [14] with the PubMLST database [15] to determine multilocus sequence types (STs). The allele profiles of unidentified STs were submitted to the MLST database of the Pasteur Institut for novel ST assignment. A minimum spanning tree was generated with PHYLOViZ using the MLST data [16]. Roary (V3.13.0) [17] was used to calculate the pangenome using the annotated assemblies in GFF3 format, which were generated by Prokka (V1.14.6) [18]. These analyses were carried out using Roar. High-quality single nucleotide polymorphisms (SNPs) were detected using the CFSAN SNP pipeline in the GalaxyTrakr (V21.09) platform [19]. SNPs were used to construct a maximum likelihood phylogeny using \RAxML (V8.2.12) [20] with the Generalized time reversible (GTR) + GAMMA distribution substitution model and 1000 rapid bootstrap iterations.

### Comparative genomic analyses using the Pathogen Detection database

The genome sequences of *L. monocytogenes* isolates were submitted to the NCBI Pathogen Detection [21] by following the standard submission guidelines provided by the Pathogen Detection database. After submission, the results of automated comparative analyses that cluster closely related isolates were examined.

## Results and discussion

### A total of 3 *L. monocytogenes* and 12 *L. innocua* MLST sequence types were detected among the studied isolates

The median genome size of 15 *L. monocytogenes* was 2.99 Mbp and the median N50 was 0.26 Mbp. The *L. monocytogenes* pangenome consisted of 3,821 genes, of which 2,254 genes were core genes detected in all studied isolates. The *L. monocytogenes* isolates represented three MLST STs, including ST 2 (87%), ST 145 (7%), and ST 18 (7%).

The median genome assembly size of the analyzed *L. innocua* isolates was 2.84 Mbp and the median N50 was 0.75 Mbp. The *L. innocua* pangenome had 4,440 genes, which included 2,434 core genes shared by all studied isolates. The 55 *L. innocua* isolates represented 12 MLST sequence types (STs). Among these, the most common ST was ST 1087 (36%), followed by ST 1619 (13%), ST 637 (13%), ST 537 (7%), ST 474 (7%), ST 1008 (5%), ST 1489 (5%), ST 492 (4%), ST 3007 (4%), ST 603 (2%), ST 1010 (2%), and one novel ST 3186 (2%). The novel ST represented a novel combination of seven allele types used for MLST typing. Specifically, ST 3186 carried a novel *bglA* allele type (i.e., 643), and the isolate from this study was the only representative of this ST. The ST 3186 belongs to the clonal complex ST-1619, which contains another ST (ST 1619) represented by 13 isolates obtained from sheep and cattle in Spain, and from cutlets in Russia.

### *Listeria monocytogenes* MLST ST 2 was detected in three regions in Ethiopia, whereas STs 145 and 18 were detected exclusively in Oromia

The *L. monocytogenes* isolates were obtained from both raw milk and pasteurized milk samples, but not cottage cheese, possibly due to *L. monocytogenes’* sensitivity to low pH or competitive exclusion by lactic acid bacteria present in the cottage cheese [22, 23]. *L. monocytogenes* positive samples were collected from 9 sites across 8 areas at 9 different time points (Fig. 1C). Among these isolates, we identified three genotypes: ST 2, ST 145, and ST 18 (Fig. 1C). The identified STs have been previously reported in other studies from various sources. For example, ST 2 was isolated from pork in Wuhan, China [24] and ST 145 was detected in ready-to-eat food, food production environments, and raw meat in Poland [25]. Furthermore, ST 18 was detected in ruminant clinical isolates and environmental samples such as in cases of sheep rhombencephalitis, bovine abortion, goat farm soil, and cattle feed bunk in Switzerland [26]. The ST 18 was also present in abortion (sheep/cattle) and enteritis (sheep) cases in Great Britain [26]. None of these STs have previously been reported in peer-reviewed literature from African countries, including the South African listeriosis outbreak, where ST 6 was the most common genotype isolated from human cases [4]. However, ST 2 and ST 145 *L. monocytogenes* isolates from multiple other African countries have been deposited in the MLST database of the Institut Pasteur. Specifically, two ST 2 isolates deposited in the MLST database were obtained from a human and a food source in Morocco, three were obtained from a human source and one from a natural environment in Tunisia, and two from a human, one from food (lentil salad), and one from an animal source in Algeria. Furthermore, the ST 145 was reported, in the MLST database, from Senegal (unknown source) and Togo (animal source). Both of these STs belong to lineage I (MLST database predicted PCR serotype IVb), which has previously been associated with human listeriosis. The distribution of STs 2 and 145 across different sources and countries indicates their ability to survive in various environments.

Among the 15 *L. monocytogenes* isolates analyzed in the current study, 13 belonged to ST 2. Remarkably, the ST 2 was detected in both raw and pasteurized milk samples collected from different collectors, processors, and producers across all three regions, suggesting its transmission through the supply chain. ST 145 and 18, on the other hand, were detected exclusively in the Oromia region. Notably, we obtained 4 isolates from pasteurized milk samples from processor P27, in both 2020 and 2021, all of which were *L. monocytogenes* ST 2. This suggests the persistence of this genotype in the milk processing facility and raises concerns about the potential role of inadequate environmental cleaning and sanitation procedures in its persistence. Additionally, this suggests potential failures in pasteurization that may contribute to the persistence of ST 2 genotype in the pasteurized milk samples. Insufficient pasteurization leading to listeriosis has occurred in the past. For instance, a notable outbreak of listeriosis occurred in 1985, stemming from contaminated Mexican-style cheese in California, USA, which led to 142 illnesses and 3 deaths. This outbreak was attributed to either inadequate pasteurization of milk or the introduction of raw milk into pasteurized milk during the manufacturing process [27].

Among the 13 isolates identified as ST 2, 11 of them were highly similar, with only 1 to 10 SNPs observed among them (Fig. 1C). However, the remaining two isolates showed a higher level of genetic variation, differing by 50 to 100 SNPs. Notably, STs 2 and 145 belong to lineage I, while ST 18 belongs to lineage II. Lineage I strains of *L. monocytogenes* are frequently associated with human clinical cases and are primarily implicated in outbreaks involving invasive disease and pose a higher risk of causing severe illness [28]. In contrast, lineage II strains are commonly found in various foods and are prevalent in environments such as soil, water, and plant material. While lineage II strains can still cause infections in humans, they have been associated with a lower incidence of listeriosis compared to lineage I isolates [28]. It is important to note that the virulence and disease-causing potential can vary among individual strains within each lineage. Furthermore, factors such as host susceptibility and food handling practices play a key role in the risk of foodborne illness [29, 30].

Through comparative genomic analyses using the Pathogen Detection database, we found that the ST 145 isolate (WGS accession number SRR19739177) belonged to the cluster PDS000003255.65 (as of August 9, 2023; please note that cluster names in the Pathogen Detection database may not remain static over time). At the time of analyses (May 17, 2023) and reporting (August 9, 2023), this cluster included a total of 84 isolates from 16 countries, including Canada, China, Poland, and the United States of America. Isolates in this cluster were obtained from diverse sources, including clinical feces, environmental swabs, and food samples (Fig. S1). This comparative genomic analysis also yielded valuable information regarding the Min-same and Min-diff. The Min-same value indicates the minimum SNP distance between our isolate and another isolate from the same source category, either clinical (human origin) or environmental (non-human origin), within the cluster. Conversely, the Min-diff value indicates the minimum SNP distance between the isolate of interest and isolates within the cluster that had been obtained from a different source. We observed a minimum difference of 3 SNPs between our food isolates compared to the isolate PDT000283214.3, which was obtained from an environmental sponge swab of a deli slicing table on October 23, 2017 in Iowa, USA. The minimum SNP difference between our isolate and the nearest clinical isolate (PDT001285134.1) was 3 SNPs. This suggests the potential for a ST 145 isolate to cause human infections, although this cannot be concluded due to the lack of epidemiological data. In the study conducted by Chen et al., a high genomic similarity was observed between outbreak-associated isolates from stone fruits and clinical isolates [31]. These isolates clustered in two distinct phylogenetic clades. In clade 1, the genetic variability between the outbreak-associated isolates and clinical isolates ranged from 0 to 2 SNPs. In clade 2, which included a different clinical isolate, the genetic variability extended from 0 to 11 SNPs.

### Distribution of *L. innocua* MLST sequence types was region-specific in Ethiopian dairy supply chain, with the exception of pan-regional presence of ST 1087

Several *L. innocua* MLST sequence types showed region-specific occurrence in Ethiopia. For instance, *L. innocua* ST 1489 isolates were found only in Amhara, and isolates of STs 1619, 603, 537, 1010, and 3186 were observed only in Oromia. Furthermore, *L. innocua* STs 492 and 3007 were detected only in SNNP region. In contrast, ST 1087 was detected in all three regions, and STs 474, 1008, and 637 were detected in two regions (Fig. 2C). Most of these STs were widely distributed and were found in various sources and environments. For example, ST 1087, ST 637, ST 603, and ST 1008 have been detected in dairy processing facilities in Austria [32]. Gana et al. reported the presence of ST 1619, ST 637, ST 1008, ST 1087, and ST 1489 at cattle farms, beef abattoirs, or retail outlets in Gauteng Province, South Africa [33]. Furthermore, in a study from South Africa, the ST 537 was detected at various levels of the meat supply chain, including butcheries, abattoirs, processing facilities, and retail outlets, and the ST 637 was specifically found in butcheries [34]. Lastly, *L. innocua* ST 1087, ST 1619, ST 637, ST 603, ST 1010, ST 537, and ST 474 were isolated from both cattle abortion cases and the farm environment in Latvia [35].

In the current study, we identified a total of 9 *L. innocua* isolates from 8 sampling sites across five areas in Amhara at 7 different time points. The ST 474 was detected in 3 raw milk samples from producers and collector in areas A and C in different years, suggesting it is endemic in Amhara (Fig. 2D). On the other hand, three raw milk samples obtained from two different producers in areas A and B contained ST 1489. However, ST 1489 was not detected at other levels of a dairy supply chain in Amhara.

Noteworthy, *L. innocua* ST 1087 was detected in pasteurized milk acquired from a retailer in area X in November 2020 (Fig. 2D). This same genotype was also found in two raw milk samples collected from a producer in area C and a collector in area D in December 2020. The detection of ST 1087 in pasteurized milk suggests two potential scenarios: either the pasteurization process was ineffective in eliminating this specific genotype, or the contamination occurred after pasteurization, possibly during packaging, handling, or storage. Previous research conducted in Norway and Sweden revealed that spoilage bacteria recontamination occurred during the filling procedure of pasteurized milk [36]. Similarly, Oltramari et al. suggested that failures in the pasteurization process or contamination during post-pasteurization processing caused the presence of *Escherichia coli* in pasteurized milk [37]. The presence of ST 1087 in pasteurized milk, along with its detection in raw milk samples, highlights the critical importance of implementing rigorous hygiene practices throughout the entire dairy supply chain, including at the farm level, to mitigate the transmission of contaminants through the supply chain. These practices include maintaining a clean farm environment and proper sanitation during milk collection and processing. The latter encompasses employing effective pasteurization processes and maintaining hygienic conditions during the handling, storage, and distribution of milk products.

In Oromia, a total of 25 *L. innocua* isolates were collected from 18 sampling sites distributed across 7 different areas at 15 different time points (Fig. 2D). These isolates represented 11 STs, among which ST 1087 was the most prevalent and was found in six areas. ST 1619 was detected in four areas, followed by ST 537 and ST 637, which were found in three and two areas, respectively. Other STs found in Oromia included STs 474, 603, 1008, 3186, and 1010, each found in one area. *L. innocua* ST 1087 was detected in different types of products, including raw milk, cottage cheese, and pasteurized milk, collected from different levels in the supply chain, including collectors, producers, processors, and retailers. Similarly, ST 1619 was found in both raw milk and pasteurized milk samples obtained from collectors, producers, processors, and retailers. In addition, a total of 3 *L. innocua* isolates belonging to ST 637 were detected in both raw milk and cottage cheese samples collected from a collector and two different producers. Lastly, ST 537 was observed in 3 raw milk and 1 pasteurized milk sample from a collector, processor, and different producers in Oromia. The identification of these genotypes in samples collected from different levels along the supply chain, from different isolation sources, and in various areas and sampling sites indicates their broad distribution in the dairy supply chain in Oromia. Again, these findings emphasize the critical importance of maintaining rigorous hygiene practices at each stage of the dairy supply chain to promote the safety of dairy products.

The greatest diversity of *L. innocua* STs was found at a milk collection level in Oromia (Fig. 2D). For example, ST 1087, 1619, and 3186 were all detected on the same day from milk samples collected from the same milk collector (i.e., P11). Furthermore, three different genotypes (STs 637, 537, and 474) were detected in samples collected on different days and years from collector P16. The presence of diverse *L. innocua* genotypes at the milk collection level in Oromia highlights the potential impact of milk collectors on the dissemination of *L. innocua* within the milk supply chain. The activities and practices of milk collectors can contribute to the introduction and spread of various *L. innocua* genotypes, which increases the risk of contaminant transmission. Therefore, it is essential to provide adequate training to milk collectors regarding proper hygiene protocols to ensure rigorous cleanliness standards are maintained throughout the milk collection process, thereby reducing the chances of contamination.

In SNNP, 21 *L. innocua* isolates were gathered from 13 sites from 11 distinct areas at 13 different time points (Fig. 2D). Among them, nine *L. innocua* isolates were obtained from pasteurized milk samples collected on different dates from a single processor (P27). These isolates belonged to four different genotypes (ST 1087, 1008, 637, and 3007). Furthermore, three of those genotypes (ST 1087, 637, and 3007) were also detected in other sample types, including raw milk and cottage cheese samples, which were collected from different producers and collectors across multiple areas in the SNNP. These findings suggest the greatest potential for contamination at the processing stage, emphasizing the need for implementation of interventions at this level in the dairy value chain.

Ultimately, this study highlights the presence of genetic diversity among *L. innocua* isolates in each region, with the identification of region-specific STs. However, it should be noted that the limited number of isolates collected from certain regions may have resulted in a bias in the discovered genotype diversity. For instance, in Amhara, we collected 9 isolates and identified 3 STs. However, with a larger sample size, it is likely that we would have discovered additional STs and found that some of the initially identified STs may not be region-specific. Therefore, further research with a more extensive sampling effort may result in a more comprehensive understanding of the genetic diversity and regional distribution of *L. innocua* isolates.

Through high quality SNP (hqSNP) analysis, we found that *L. innocua* isolates within individual STs (i.e., STs 1087, 1008, 637, 1489, 3007, 492, 537, and 474) showed low intra-ST genetic variation with only 0–10 SNP differences observed among isolates. However, *L. innocua* isolates belonging to the ST 1619 exhibited a greater diversity, as reflected by 0 to 100 SNP difference among isolates (Fig. 2D).

## Conclusion

In conclusion, the majority of *L. monocytogenes* isolates obtained from raw and pasteurized milk collected in Ethiopia belong to STs 2 and 145. These two STs belong to lineage I, and lineage I strains of *L. monocytogenes* have been frequently associated with human clinical cases and are primarily implicated in outbreaks involving invasive disease and pose a higher risk of causing severe illness. The risk for listeriosis associated with the consumption of milk contaminated with *L. monocytogenes* lineage I isolates may be reduced by heat treatment of milk prior to consumption.

### Electronic supplementary material

Below is the link to the electronic supplementary material.

**Supplementary Material 1: Fig. S1.** Phylogenetic tree for cluster PDS000003255.65 isolates obtained from the NCBI Pathogen Detection. The isolate from this study is highlighted in red

## Data Availability

The datasets analyzed in the current study are available in the NCBI Sequence Read Archive (SRA) repository under the accession number PRJNA357724.

## Abbreviations

*L. monocytogenes*: *Listeria monocytogenes*
*L. innocua*: *Listeria innocua*
SNP: Single nucleotide polymorphism
WGS: Whole-genome sequencing
SNNPR: Southern Nations, Nationalities, and Peoples’ Region
