# Supplementary material for: Genomic characterization of Listeria monocytogenes and Listeria innocua isolated from milk and dairy samples in Ethiopia
Source: BMC Genom Data. 2024 Jan 31;25:12. doi: 10.1186/s12863-024-01195-0 (PMC10829315; doi:10.1186/s12863-024-01195-0)
Supplement: Supplementary file 1 — Supplementary Material 1: Fig. S1. Phylogenetic tree for cluster PDS000003255.65 isolates obtained from the NCBI Pathogen Detection. The isolate from this study is highlighted in red [file 12863_2024_1195_MOESM1_ESM.docx]

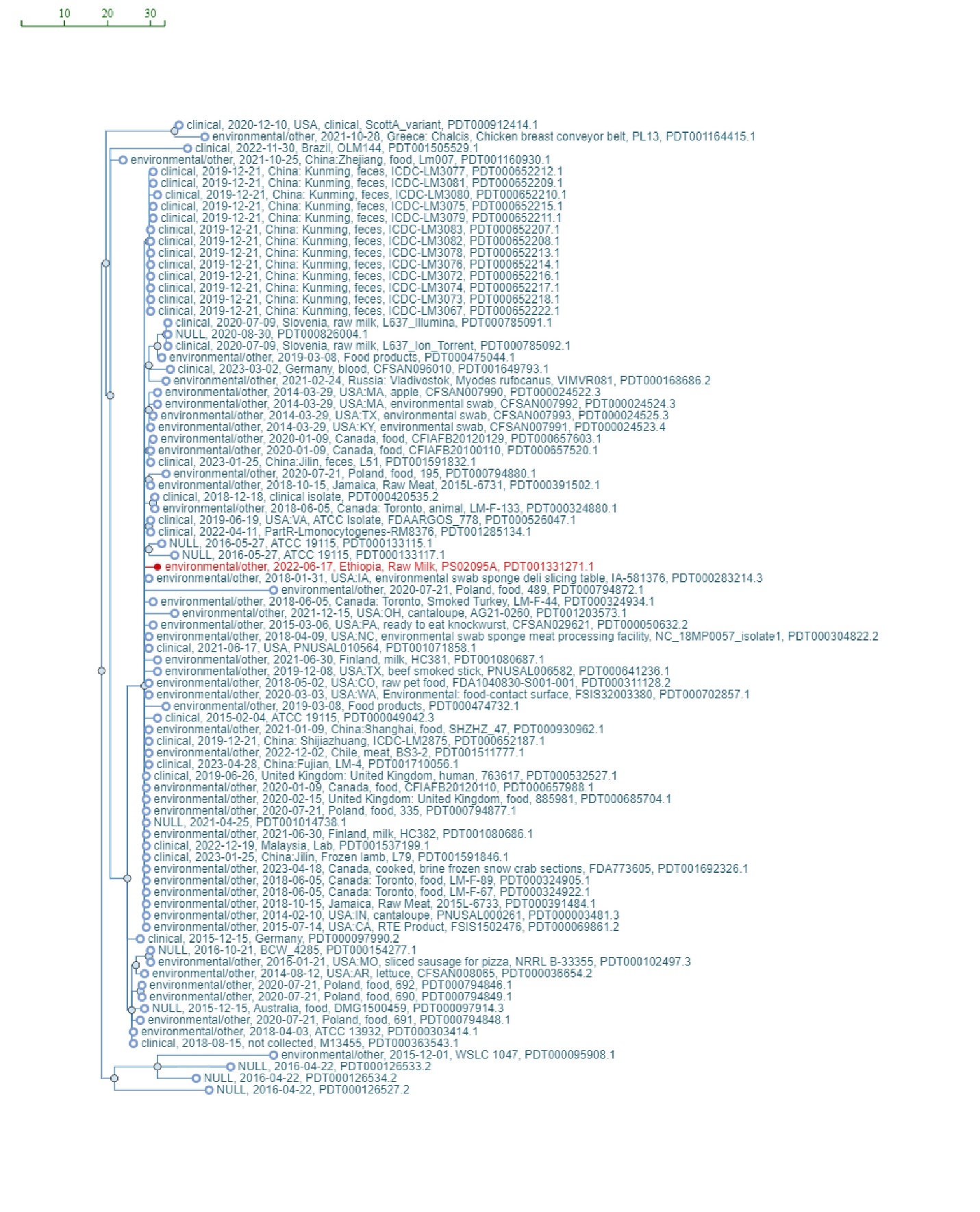


Fig. S1. Phylogenetic tree for cluster PDS000003255.65 isolates obtained from the NCBI Pathogen Detection. The isolate from this study is highlighted in red.
